# Anterior versus posterior approach laparoscopic radical cystectomy: a retrospective analysis

**DOI:** 10.1186/s12957-018-1547-7

**Published:** 2019-01-07

**Authors:** Shiyu Tong, Zhenyu Yang, Xiongbin Zu, Yuan Li, Wei He, Yangle Li, Xiheng Hu, Cheng Zhao, Minfeng Chen, Lin Qi

**Affiliations:** Department of Urology, Xiangya Hospital, Central South University, 87 Xiangya Rd, Changsha, 410008 Hunan People’s Republic of China

**Keywords:** Bladder cancer, Bladder suspension, Anterior approach laparoscopic radical cystectomy, Complication

## Abstract

**Objective:**

To investigate the mortality, operation time, cystectomy time, and complications of anterior approach laparoscopic radical cystectomy (ALRC) in Asian males in comparison with posterior approach laparoscopic radical cystectomy (PLRC).

**Materials and methods:**

One hundred forty-seven male patients with bladder cancer (cT2-3NxM0) in our hospital from May 2011 to January 2018 having undergone laparoscopic radical cystectomy were studied, including 68 patients in PLRC group and 79 patients in ALRC group. Baseline patient characteristics, operative and postoperative characteristics, and postoperative complications were retrospectively collected and analyzed between the two groups.

**Results:**

Patients in these two groups exhibited similar baseline characteristics (*p* > 0.05). Compared with PLRC group, ALRC group required similar operation time (317.3 ± 40.9 vs 321.9 ± 37.5) and cystectomy time (64.8 ± 8.7 vs 65.6 ± 14.0). The ALRC group required less cystectomy time (67.8 ± 10.1 vs 77.4 ± 14.9) when patients’ BMI > 24 or patients had large total tumor and blood clot volume (> 160 cm^3^). Also, estimated blood loss (EBL) of ALRC group was significantly less than that of PLRC group (477.8 ± 97.4 vs 550.4 ± 99.9). There existed no significant differences between the PLRC and ALRC groups in postoperative characteristics and complications.

**Conclusion:**

This study revealed that ALRC required less cystectomy time for patients with higher BMI and larger tumor, suggesting less blood loss and similar perioperative complications. ALRC is recommend for male patients, of which BMI > 24 or total tumor and blood clot volume > 160 cm^3^.

## Introduction

Bladder cancer (BCa), characterized by high risk of recurrence and mortality, is a serious health risk worldwide, with an estimated 430,000 new cases in 2012 [1]. BCa is common in elder individuals and strongly associated with smoking exposure [2, 3]. The incidence of BCa has been rising in China with the population aging, and BCa ranks the first in the urinary malignant tumor with an incidence of 69.7/100,000 [4]. Accordingly, the treatment for bladder cancer is of huge importance.

Radical cystectomy (RC) is the standard management of non-metastatic, muscle-invasive BCa [5]. Though open radical cystectomy (ORC) is a universally accepted gold standard for treating muscle-invasive, organ-confined BCa, minimally invasive surgical techniques, such as laparoscopic radical cystectomy (LRC) and robot-assisted laparoscopic radical cystectomy (RALRC), have been rapidly accepted for treating BCa. Compared with ORC, these new techniques with small incisions are considered to cause less blood loss and shorter hospital stay, while yielding equivalent oncologic and functional outcomes [6–8]. However, LRC has not been widespread because of the technical challenges of the procedure. The process of LRC is long and complicated, requiring detailed knowledge of pelvic anatomy. LRC is really challenging for male patients with higher BMI and larger tumors, even for an experienced surgical team. As the working space in the narrow pelvis would be smaller, laparoscopic visualization would be limited.

The LRC techniques for male fat patients or male patients with larger tumor and blood clot have been rarely studied. To make better laparoscopic visualization and bigger working space, posterior approach laparoscopic radical cystectomy (PLRC) was modified in 2011, and bladder suspension was performed during the laparoscopic radical cystectomy. To compare PLRC with anterior approach laparoscopic radical cystectomy (ALRC), 147 male patients who underwent PLRC or ALRC in our hospital from May 2011 to January 2018 in this study were retrospectively analyzed, and our experience of ALRC was reported.

## Materials and methods

### Patients

This study was approved by the ethics board of Xiangya Hospital, Central South University, in Changsha, China. All the participants were informed that their clinical information may be applied in later clinical study when they entered hospital. Also, their written informed consents were given. Between May 2011 and January 2018, there were 157 male patients in our hospital, with high-grade bladder urothelial carcinoma, of which 72 and 85 patients are in the PLRC and ALRC group respectively; we failed to follow up 4/72 patients in PLRC group and 6/85 patients in ALRC group. Thus, these patients were excluded, and the medical records of the 147 patients retrospectively were collected and analyzed from our bladder cancer database. We made the technique rotation from month to month. All patients have high-grade bladder urothelial carcinoma (cT_2-3_N_X_M_0_) in line with TMN classification of the Union for International Cancer Control [9]. All patients had primary tumors, and none of the patients had received bladder surgeries, radiotherapy, or chemotherapy before.

Patients were evaluated in accordance with the American Society of Anesthesiologists (ASA) physical status classification system. Each patient underwent preoperative examination including routine laboratory test, echocardiography, chest radiography, lung function test, computerized tomography, or magnetic resonance imaging of urinary system. Common comorbidities were recorded, including diabetes mellitus, coronary artery disease, hypertension, chronic obstructive pulmonary disease, and other chronic diseases. Patients began semi-liquid diet 48 h before surgery, liquid diet 24 h before surgery, and underwent bowel preparation with enema and oral sodium phosphates solution 12 h before surgery. All patients got broad-spectrum systemic antibiotic intravenously during the induction of anesthesia. Same laparoscopic instruments and devices from KARL STORZ were used in both groups. All the operations and perioperative management were performed by the same experienced surgical team. Three surgeons (MC, LQ, and XZ) performed the operations. MC performed 30 and 35, LQ performed 22 and 24, and XZ performed 16 and 20 in PLRC and ALRC respectively. All the surgeons are very experienced and perform more than 25 cases of laparoscopic radical cystectomy annually. Follow-up studies were conducted with ultrasonography and CT scan at 3 and 9 months postoperatively.

### Parameters and endpoint

The baseline patient characteristics included age, BMI, hemoglobin (Hb), total tumor and blot clot volume, comorbid conditions, and ASA score. Total tumor and blot clot volume were measured with CT scan. Operative characteristics included operation time, cystectomy time, estimated blood loss (EBL), and transfusion needed, while postoperative characteristics included Hb, time to liquid intake, time to exsufflation, and hospital stay after surgery. Operation time was defined as anesthesia from the beginning to the end, and cystectomy time was defined as procedure from starting mobilization to bladder removed completely. Intraoperative complication includes rectal injury and external iliac vein injury. Complications that occurred within 90 days after surgery were considered early complications, which included ileus, deep vein thrombosis, pyelonephritis, infection, obturator nerve paresis, and wound dehiscence [10]. The complication occurring within 90 days or later after surgery were defined as late complications, including ileus, ureteral stricture, and incisional hernia [10].

### Statistical analysis

Chi-square test and Student’s *t* test were performed to compare categorical and parametric data respectively between the two groups. Parametric data is denoted as mean ± SD, and categorical data is presented as frequency (%). Linear regression analysis was conducted to assess the relationship between cystectomy time and total tumor and blood clot volume. Significant differences were considered when *p* < 0.05. Statistical analysis was conducted with the Statistical Package for Social Scientists, version 22.0 (IBM Inc).

### Laparoscopic radical cystectomy

The basic laparoscopic radical cystectomy procedures were performed according to Campbell-Walsh Urology [11], A five-port fan-shaped trans-peritoneal approach was employed for LRC. The camera port was placed just below the umbilicus. The residual four ports were placed under endoscopic control after the establishment of the pneumoperitoneum. A dorsal supine position with a 20–25° Trendelenburg position was used to keep the bowel from the pelvis during surgery. Surgery was performed by visualizing the pelvis and releasing adhesions of the sigmoid colon to find the anatomical landmarks in the pelvis, such as obliterated umbilical arteries, obliterated urachus, spermatic cord, and vas deferens. The standard lymphadenectomy was performed in all cases [12]. After laparoscopic cystectomy, urinary diversion, bricker operation, or orthotopic neobladder, was conducted according to patients’ preferences.

### Posterior and anterior approach laparoscopic radical cystectomy

**Fig. 1:**
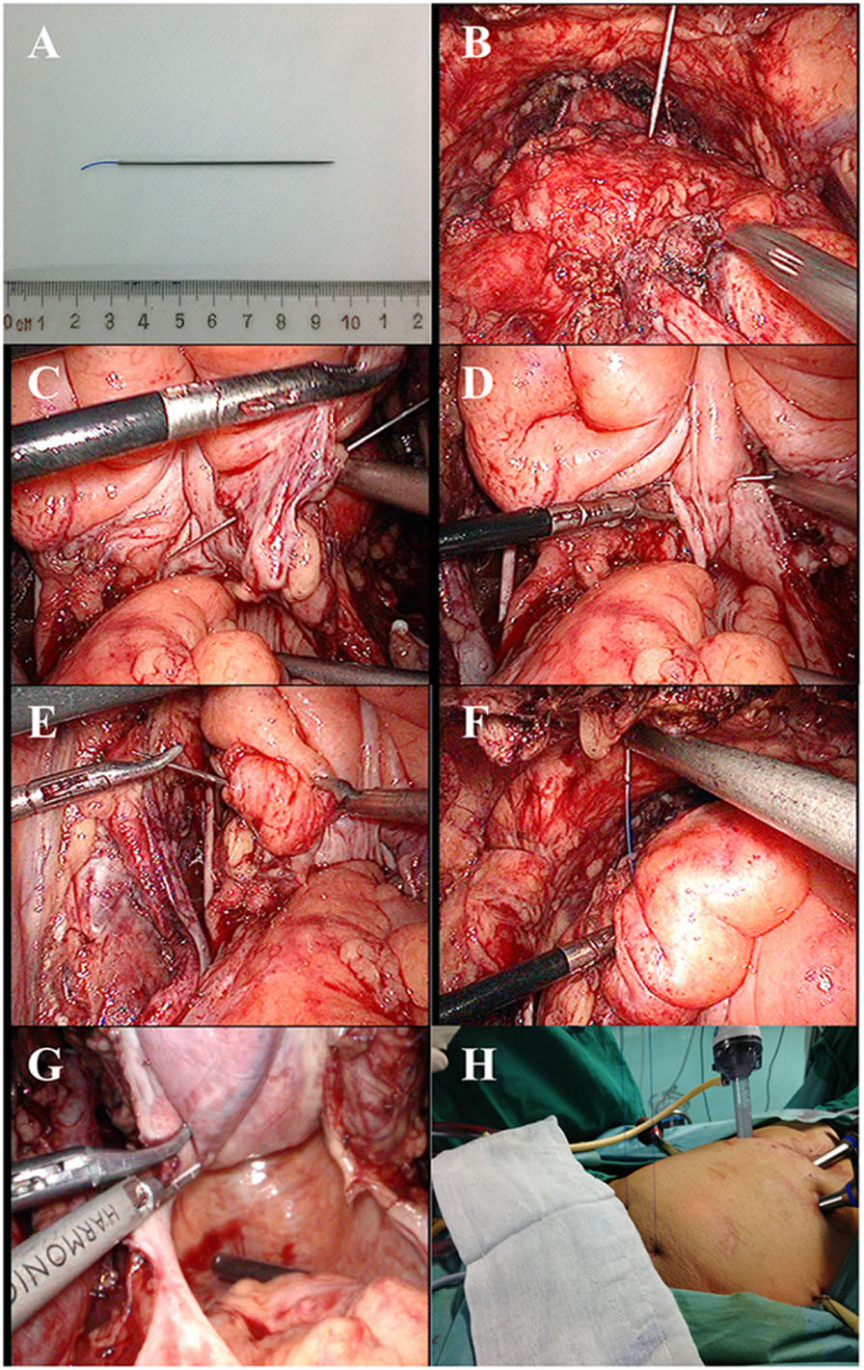
Bladder suspension technique. **a** 2-0 synthetic non-absorbable (Polyamid 6, Ethilon*II) suture and needle. **b** The needle was puncturing into the abdomen. **c** The needle was puncturing through the right side bladder wall. **d** The needle was puncturing through the lower posterior bladder wall. **e** The needle was puncturing through the left side bladder wall. **f** The needle was puncturing through the abdominal wall out of abdomen. **g** The extended space of vesicorectal fossa with the help of bladder suspension. **h** View of the suture outside before knotting

The posterior approach laparoscopic radical cystectomy is a conventional LRC, the posterior plane was developed first, and the whole operation procedure was performed according to Campbell-Walsh Urology [11]. The anterior approach laparoscopic radical cystectomy was performed as follows. The anterior plane was first developed distally towards the prostate. Both puboprostatic ligaments were mobilized and dissected, and the Santorini venous plexus was ligated. Subsequently, the bladder was suspended towards the abdominal wall with a 2-0 synthetic non-absorbable suture (Fig. 1a). The laparoscopy observation showed that nearly half of the needle was punctured into the abdominal, 3 cm above the pubic symphysis, and about 2 cm right to the midline (Fig. 1b). The needle was punctured through the right side of bladder wall, then intermittently through the posterior and left side, puncturing out to the symmetrical position of the abdominal wall (Fig. 1c–f). The needle should be avoided from penetrating the bladder wall, and the suture should be limited in the superficial peritoneum. After puncturing out the abdominal wall (Fig. 1g), the end of this suture was knotted with tension. The vesicorectal fossa could extend to the fullest after bladder suspension was completed (Fig. 1h). After bladder suspension, the posterior plane was developed. Next, the procedures of handling the posterior and lateral planes were the same as those of PLRC. After the posterior and lateral bladder attachments were released, the bladder was released from the anterior abdominal wall. The attachments of the prostatic apex to the pelvic floor were released, and the urethral catheter was removed. After the dissection of Santorini venous plexus, the urethra was dissected.

## Results

### Baseline patient characteristics

In general, 147 patients were recruited here, including 68 patients who underwent PLRC and 79 patients proceeded with ALRC. Patients in the PLRC group and ALRC group had comparable baseline characteristics, including age, BMI, Hb, total tumor and blood clot volume, comorbid conditions, and ASA score (Table 1).

**Table 1:** Baseline patient characteristics

Characteristics	PLRC (*n* = 68)	ALRC (*n* = 79)	χ^2^/*t*	*p* value
Age, median (range)	57 (44–81)	59 (47–80)	0.629*	0.531
BMI (kg/m^2^)	21.7 ± 3.1	22.3 ± 3.3	0.333*	0.730
Hb (g/L)	121.2 ± 11.7	120.6 ± 10.5	0.330*	0.742
Total tumor and blood clot volume	179.0 ± 72.2	186.0 ± 66.8	0.697*	0.487
Comorbid conditions				
Diabetes mellitus	15 (22.1%)	20 (25.3%)	0.214	0.644
Hypertension	20 (29.4%)	22 (27.8%)	0.044	0.834
Coronary heart disease	5 (7.4%)	4 (5.1%)	0.333	0.564
COPD	3 (4.4%)	4 (5.1%)	0.034	0.853
Other chronic diseases	3 (4.4%)	2 (2.5%)	0.393	0.531
ASA score				
2	52 (76.4%)	60 (75.9%)	0.083	0.773
3	16 (23.6%)	19 (24.1%)		

*t value

### Operative and postoperative characteristics

One patient in the PLRC group was transferred to open surgery, and no patients in the ALRC groups were transferred to open surgery. Operative and postoperative characteristics of the two groups are shown in Table 2. Estimated blood loss (EBL) of the ALRC group was significantly lower than that of the PLRC group (477.8 ± 97.4 vs 550.4 ± 99.9). Also, less patients in ALRC group needed transfusion (Table 2). The Hb after surgery in ALRC group was a little higher than the PLRC group (102.1 ± 11.6 vs 98.1 ± 12.5). There were no significant differences between the two groups in operation time, cystectomy time, time to liquid intake, time to exsufflation, and hospital stay after surgery.

**Table 2:** Operative and postoperative characteristics

Characteristics	PLRC (*n* = 68)	ALRC (*n* = 79)	χ^2^/*t*	*p* value
Operation time (min)	321.9 ± 37.5	317.3 ± 40.9	0.702*	0.484
Operation time (min, volume > 160 cm^3^)	340.8 ± 25.2 (*n* = 37)	320.7 ± 42.6 (*n* = 51)	2.626*	0.010
Operation time (min, volume ≤ 160 cm^3^)	296.4 ± 36.5 (*n* = 31)	310.8 ± 37.7 (*n* = 28)	1.434*	0.157
Cystectomy time (min)	65.6 ± 14.0	64.8 ± 8.7	0.375*	0.708
EBL (ml)	550.4 ± 99.9	477.8 ± 97.4	4.458*	< 0.001
Hb (g/L)	98.1 ± 12.5	102.1 ± 11.6	1.993*	0.048
Transfusion needed	21 (25.0%)	13 (27.8%)	4.277	0.039
Time to liquid intake (day)	1.9 ± 0.6	2.0 ± 0.7	1.167	0.245
Time to exsufflation (day)	3.2 ± 0.6	3.4 ± 0.8	1.516	0.131
Hospital stay after surgery (day)	7.7 ± 1.5	7.5 ± 1.7	0.733	0.465
Conversions to open procedure	1	0	1.170	0.279

*t value

**Fig. 2:**
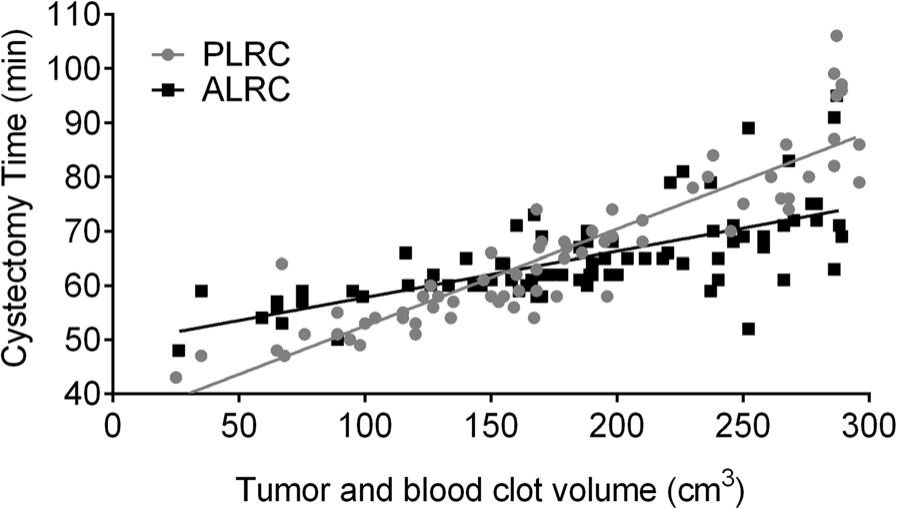
The linear regression analysis between cystectomy and total tumor and blood clot volume

When patients were sub-grouped based on BMI, cystectomy time was different between sub-groups though the operation time (317.3 ± 40.9 vs 321.9 ± 37.5) and cystectomy time (64.8 ± 8.7 vs 65.6 ± 14.0) were similar between the two groups. When BMI was less than 18.5, the PLRC group took less cystectomy time compared with the ALRC (52.5 ± 6.5 vs 60.3 ± 6.2). When BMI was ranged from 18.5 to 24, cystectomy time was similar between the two groups. It took longer cystectomy time for the PLRC group when BMI was greater than 24 (77.4 ± 14.9 vs 67.8 ± 10.1) (Table 3). Liner regression analysis was conducted to access the relationship between cystectomy time and total tumor and blood clot volume. In both PLRC (*y =* 0.1787*x* + 34.66, *r*^2^ = 0.8225, *p* < 0.0001) and ALRC (*y =* 0.0851*x* + 49.29, *r*^2^ = 0.4459, *p* < 0.0001), cystectomy time were correlated significantly positively with total tumor and blood clot volume. Both PLRC and ALRC required similar cystectomy time when total tumor and blood clot volume was about 160 cm^3^. Compared with ALRC, PLRC required less cystectomy time and operation time when total tumor and blood clot volume was less than 160 cm^3^, and PLRC required more cystectomy time and operation time when total tumor and blood clot volume was larger than 160 cm^3^ (Table 2, Fig. 2).

**Table 3:** Cystectomy time based on BMI

BMI	PLRC (min, *n*)	ALRC (min, *n*)	*t*	*p* value
BMI ≤ 18.5	52.5 ± 6.5 (12)	60.3 ± 6.2 (16)	3.216	0.004
18.5 < BMI ≤ 24	64.7 ± 11.3 (40)	65.2 ± 8.3 (44)	0.223	0.825
BMI > 24	77.4 ± 14.9 (16)	67.8 ± 10.1 (19)	2.264	0.030

### Intraoperative and postoperative complications

There were no deaths in the two groups during the perioperative period. Intraoperative and postoperative complications of the two groups are listed in Table 4. Two patients underwent rectal injury and another patient got external iliac vein injury in the PLRC group during surgery, and one patient underwent external iliac vein injury in the ALRC group. Ileus was considered the most common early postoperative complication, and there existed no significant difference between the PLRC and ALRC group. Also, there were no significant differences between the two groups in the incidence of other early complications, including deep vein thrombosis, pyelonephritis, infection, obturator nerve paresis, and wound dehiscence. Likewise, the incidence of late complications including ileus, ureteral stricture, and incisional hernia was comparable.

**Table 4:** Perioperative complications

Complications	PLRC (*n* = 68)	ALRC (*n* = 79)	*χ* ^2^	*p* value
Intraoperative				
Rectal injury	2 (2.9%)	0 (0.0%)	2.356	0.125
External iliac vein injury	1 (1.5%)	1 (1.3%)	0.011	0.915
Early complications (≤ 90 days)				
Ileus	2 (2.9%)	3 (4.0%)	0.082	0.775
Deep vein thrombosis	2 (2.9%)	2 (2.5%)	0.023	0.879
Pyelonephritis	1 (1.5%)	2 (2.5%)	0.206	0.650
Infection	1 (1.5%)	1 (1.3%)	0.011	0.915
Obturator nerve paresis	1 (0.0%)	0 (0.0%)	1.170	0.279
Wound dehiscence	1 (1.5%)	0 (0.0%)	1.170	0.279
Late complications (> 90 days)				
Ileus	2 (2.9%)	1 (1.3%)	0.513	0.474
Ureteral stricture	1 (1.5%)	1 (1.3%)	0.011	0.915
Incisional hernia	1 (1.5%)	0 (0.0%)	1.170	0.279

## Discussion

With the rapid development of laparoscopic techniques, LRC has become an alternative procedure rather than the open radical cystectomy [13]. Besides, LPC is associated with less blood loss and shorter hospital stay and no significant differences in oncologic outcomes [6–8]. The procedure of LPC is complicated, time-consuming, difficult to grasp, and challenging to laparoscopy-naive surgeons [14, 15]. A large number of studies focused on the technique improvement of LRC, such as the modifications about pelvic lymph node dissection or urinary diversion [16, 17]. The difficulty of laparoscopic cystectomy would increase when working space and vision field were very limited in the pelvis. The present study was to assess the technical modification of laparoscopic cystectomy to make laparoscopic visualization and working space larger.

Compared with female, the pelvis of male is relatively narrower and longer [18], and the skeleton of Asians are generally smaller than the Westerns [19]. These make it even more difficult for surgeons to perform LRC in Asian male patients. In particular, the seminal vesicle and the posterior surface of the prostate become very difficult due to limited vision and working space. To expose the vesicorectal fossa, the posterior plane was developed distally towards the prostate first, usually with the assistant’s devices, pull the bladder upwards, and stretch the sigmoid colon towards the head. Besides, a catheter is indwelled routinely preoperative to keep the bladder empty to make the working space largest. For Asian males especially those with large tumor and blood clot, however, these mentioned methods would be still insufficient. The bladder would maintain a certain degree of filling state, and it was hard to produce adequate working place and make the cystectomy more difficult.

In the ALRC procedure, the anterior plane was developed first distally towards the prostate. Then, the bladder was suspended using a 2-0 polyamide stitch towards the abdominal wall (Fig. 1), to reveal the vesicorectal fossa. Even in the cases with large tumor and blood clot, the visualization and working place were excellent. Therefore, the Denonvilliers’ fascia was easily encountered at the level of the prostate-vesicular junction, and this fascia can be incised under direct vision, allowing the posterior plane to be developed further distally in a safer way, compared with PLRC. The rectum was less likely to be injured when dissecting the tissue between the prostate and the anterior rectal surface under direct vision. Moreover, for those younger male patients wanted to retain erectile function, laparoscopic nerve-sparing radical cystectomy had been performed with the same principles of open procedure, using ultrasound scalpel to dissect tissue within the prostate fascia to preserve these nerve fibers.

In the ALRC group, bladder suspension technique provided better surgical exposure and clearer anatomical landmarks, which resulted in much more smooth operation procedures. According to the results, though the average cystectomy time was similar for all patients between the two groups (64.8 ± 8.7 vs 65.6 ± 14.0), the ALRC group required less time for cystectomy when patients’ BMI was greater than 24 (67.8 ± 10.1 vs 77.4 ± 14.9). Also, when total tumor and blood clot volume was larger than 160 cm^3^, it took less time for the ALRC group (Fig. 2). EBL of the ALRC group was significantly lower than that of the PLRC group (477.8 ± 97.4 vs 550.4 ± 99.9). Better visualization and larger working place played important roles regarding the blood loss. Besides, rectal injury was a common complication and it happened primarily due to the bloody working field between the prostate and the anterior rectal surface. Bladder suspension helped to expose vesicorectal fossa fully and avoid rectal injury. No rectal injury was found here in the ALRC group (Table 4), while there were two cases in the PLRC group.

LRC should be limited to experienced and skilled laparoscopic urologists. The working space in the pelvis and proper laparoscopic visualization are vital for LRC. The bladder lies deeply in the pelvis with a limited space. For female patients, the operative field may be fully revealed by adjusting the surgical posture to a hyperextension lithotomy position, while it remains hard to achieve good better visualization for male patients. In the meantime, there was no effective method reported in the literature to achieve better visualization except the traction of the assistants’ devices. Here, a safe and effective alternative technique was reported in detail to achieve better visualization in laparoscopic cystectomy for male patients.

## Conclusion

To sum up, the technique of bladder suspension applied in ALRC is simple and effective to provide better visualization and larger working space. Compared with PLRC, it takes less time of laparoscopic cystectomy for patients with greater BMI or larger total tumor and blood clot volume, and patients also have less blood loss. The technique of bladder suspension is verified to be suitable for Asian male BCa patients, especially those with greater BMI or larger tumor and blood clot within the bladder.
